# Towards patient-centered research in pregnancy-associated breast cancer: creating a science agenda through a priority-setting-partnership

**DOI:** 10.1016/j.breast.2026.104808

**Published:** 2026-05-15

**Authors:** Carsten F.J. Bakhuis, Anna S. Koning, Britt B.M. Suelmann, Inge O. Baas, Janna W. Nijkamp, Kirsten Coutinho-Steentjes, Pleun A. Gilsing-Doreleijers, Manuela Guerra, Aaltje G.H. Hoppentocht, Elsemieke Kaashoek-de Jong, Nicol Nijholt, Roselien van der Wielen, Jan Ewout Ruiter, Sabine C. Linn, Stefan Preković, Paul J. Van Diest, Elsken van der Wall, Carmen van Dooijeweert

**Affiliations:** a Department of Pathology, University Medical Center Utrecht, Utrecht, the Netherlands; b Department of Medical Oncology, University Medical Center Utrecht, Utrecht, the Netherlands; c Department of Gynecologic Oncology, Netherlands Cancer Institute, Amsterdam, the Netherlands; d Department of Gynecology, University Medical Center Utrecht, Utrecht, the Netherlands; e Patient Representative for the MOTHER Project, University Medical Center Utrecht, Utrecht, the Netherlands; f Leren Filosoferen, Castricum, the Netherlands; g Department of Medical Oncology, Netherlands Cancer Institute, Amsterdam, the Netherlands; h Center for Molecular Medicine, University Medical Center Utrecht, Utrecht, the Netherlands; i Department of Internal Medicine, Radboud University Medical Center, Nijmegen, the Netherlands

**Keywords:** Breast cancer, Pregnancy, Patient involvement

## Abstract

**Background:**

Pregnancy-associated breast cancer (PABC) is a rare yet increasingly prevalent condition, characterized by diagnostic delay, distinct tumor biology, and complex clinical and psychosocial challenges that are insufficiently addressed within contemporary breast cancer research agendas. We aimed to develop a patient-centered research agenda for PABC through a Priority Setting Partnership (PSP), bringing together patients, clinicians, and researchers to identify and prioritize key unanswered research questions.

**Methods:**

A PSP was established comprising patients with PABC, healthcare professionals, and researchers from the Netherlands. Four focus group sessions were conducted and facilitated by an independent moderator using Socratic dialogue techniques to elicit lived experiences. Research questions were subsequently submitted and discussed, followed by prioritization through a structured weighted voting process involving both focus group participants and a broader sounding board.

**Results:**

The focus group included seven patients, four healthcare professionals, and three researchers. The sounding board consisted of 12 patients and four clinicians. Fourteen research questions were identified and prioritized using a weighted voting system. Although some priorities differed between patient participants and healthcare/research professionals, this process resulted in a consensus-based top 10 PABC research agenda, alongside five key signals aimed at improving clinical care.

**Conclusions:**

This PSP identified critical evidence gaps across biological, clinical, and psychosocial domains in PABC. The resulting research agenda underscores the need for multidisciplinary and patient-informed research approaches to advance knowledge and improve care for women affected by PABC and their families.

## Highlights

• Pregnancy-associated breast cancer (PABC) is rare but increasingly prevalent.
• Here, patients and professionals jointly set up a PABC research agenda.
• Participants ranked research needs differently but reached shared priorities.
• This agenda offers a clear path for future studies to improve PABC care.

## 1 Introduction

Pregnancy-associated breast cancer (PABC) constitutes a biological and clinical paradox: a malignancy emerging during or shortly after a physiological state marked by profound endocrine and immunological changes. PABC is defined as breast cancer diagnosed during pregnancy (PrBC) or within the years following childbirth (postpartum breast cancer, PPBC) [1]. Although rare, PABC is becoming increasingly relevant as breast cancer incidence among young women rises and childbearing is increasingly delayed to later reproductive years [2,3].

Clinically, PABC is associated with delayed diagnosis, advanced stage at presentation, and complex therapeutic dilemmas balancing maternal and fetal outcomes [[4], [5], [6], [7]]. Beyond these challenges, accumulating evidence suggests that PABC tumors differ substantially from those in age-matched non-PABC patients in biology and behavior [[8], [9], [10], [11], [12], [13]] Yet the mechanisms underlying these differences remain poorly understood.

Importantly, PABC is not only a biological entity, but a lived experience unfolding at a uniquely vulnerable life stage. Women diagnosed during pregnancy or postpartum require clinical, psychosocial and informational needs that differ substantially from those of the ‘general’ breast cancer population. PABC patients comment that these needs are insufficiently addressed in existing research agendas, since these primarily focused on areas such as breast cancer surgery and postmastectomy reconstruction [[14], [15], [16], [17]]. Likewise, the extensive 2013 research agenda for young women (<40 years) with breast cancer did not incorporate the PABC-specific perspective [18]. Consequently, despite recognized scientific gaps spanning tumor biology, clinical management, and patient-centered care, a coordinated and structured roadmap to guide future PABC research has not yet been defined.

The “multidisciplinary foundation with- and for patients with pregnancy-associated breast cancer” (MOTHER) Project seeks to address these gaps by developing a comprehensive research agenda for PABC through a Priority Setting Partnership (PSP). A PSP unites patients, clinicians, and researchers to identify and reach consensus on the most important unanswered questions, providing a structured framework for a shared research agenda [[19], [20], [21], [22]]. This approach ensures that future research reflects both the lived experiences of those affected and the clinical uncertainties faced by healthcare professionals.

By integrating diverse perspectives and systematically identifying the areas of greatest need within the field of PABC, this study aims to guide funding bodies, research teams, and policy makers toward the questions most critical for improving outcomes and quality of life of women diagnosed with PABC.

## 2 Methods

### 2.1 Participant selection

A focus group was established comprising patients, healthcare professionals, and researchers with established PABC expertise. Healthcare professionals included three medical oncologists and one gynecologist, while researchers were affiliated with the two main Dutch PABC research initiatives: the Dutch Nationwide PABC Cohort (UMC Utrecht) and the INCIP registry (Netherlands Cancer Institute – Antoni van Leeuwenhoek Hospital). Given the small scale of this PSP, no steering committee was set up, with the participating researchers functioning as study coordinators.

Patients self-referred following announcements by national patient organizations (*Borstkanker Vereniging Nederland* and *Stichting Ster(k)*) and via clinicians participating in regional tumor boards. Due to high interest (approximately 30 applicants), participants were divided into a focus group and a sounding board. The focus group included patients diagnosed with PrBC or PPBC with a varying time since diagnosis, representing both recent and longer-term treatment trajectories. Remaining applicants and additional clinicians with experience in the treatment of PABC patients joined the sounding board.

### 2.2 Sessions

All sessions were conducted in Dutch. Four focus group sessions were held under the guidance of a professional facilitator employing Socratic dialogue techniques [23]. Here, we used lived experiences of each participant to jointly identify knowledge gaps and to create consensus. The first session established criteria for high-quality research questions. Subsequently, patients and healthcare professionals of both the focus group and sounding board were invited to submit research questions, which were discussed, reformulated and pre-selected during the second session of the focus group. Upfront, literature searches were performed by the participating researchers for each question to check that the concerning topics were indeed not answered yet.

Participants from both the focus group and sounding board then prioritized the submitted research questions using a weighted voting system, where all participants had a total of 15 “tokens” which they could distribute freely over the questions. Votes were aggregated to produce a draft top-10 agenda, which was reviewed in a third focus group session. The final agenda was presented to patient organizations in the concluding fourth session.

In addition, participants were able to submit 'signals' alongside the formal research agenda. These were defined as concrete calls-to-action for improvement of clinical care that are addressable through practice change or policy, rather than new scientific research. These calls-to-action were discussed as well in the second- and third sessions for their formulations, but were not prioritized.

### 2.3 Ethical and regulatory aspects

The project was reviewed by the local research quality coordinator, who waived the need for formal ethical approval. All patients provided written informed consent and could withdraw at any time. Focus group participants were invited to review the manuscript and were offered co-authorship given their major contribution to this project, with the understanding that this would compromise their anonymity towards the funding source. Travel expenses for the two in-person sessions were reimbursed, and participants received a €25,- voucher per session in accordance with our institutional policy for patient participation.

### Funding

This study was funded by a dedicated grant from Gilead Sciences. The funder had no role in study design, data collection or analysis, agenda setting, manuscript preparation, or publication decisions, and had no access to participant identities or study content.

## 3 Results

This PSP was reported in accordance with the REPRISE guidelines [24]. A total of 10 patients were initially included in the focus group. One patient was excluded due to non-response to follow-up e-mails, whilst another patient withdrew after disease progression, both before the first session. A third patient discontinued after the first session, as she found this was more energy consuming than she anticipated upfront. Baseline characteristics of all participating patients, healthcare providers, and researchers are described in Table 1. Through the sounding board, close contact was held with an additional 12 patients and four healthcare professionals.

**Table 1:** Baseline characteristics of the focus group participants.

Participants	PABC	Time since diagnosis	Treatment status/intent
Patient A	PrBC	<5 Years	Completed, curative intent
Patient B	PrBC	<5 Years	Completed, curative intent
Patient C	PrBC	5-10 Years	Completed, curative intent
Patient D	PrBC	5-10 Years	Completed, curative intent
Patient E	PPBC	<5 Years	Ongoing, life-prolonging
Patient F	PPBC	<5 Years	Ongoing, curative intent
Patient G	PPBC	<5 Years	Completed, curative intent

Participants	Years of experience
Oncologist A	30-40 Years
Oncologist B	10-20 Years
Oncologist C	10-20 Years
Gynecologist	10-20 Years
Researcher A	5-10 Years
Researcher B	3-5 Years
Researcher C	<3 Years

In the first session of the focus group, two criteria were established for research questions to be included in the science agenda.

1. The question is relevant to either PABC patients as a group, or the question is relevant for individual PABC patients.
2. The question can be answered through scientific research.

After duplicate removal, a total of 32 questions were submitted. During the second focus group session, overlapping and related questions were consolidated into 14 broader research questions through merging, without discarding any individual submission (Supplemental Table S1). After prioritization (Supplemental Table S2), the top 10 was selected as the science agenda, presented in Table 2. The questions higher on the agenda received the highest priority according to this priority-setting partnership. Some topics were of special interest to patients (Table 2, questions 2, 8, 9 and 10), whilst the positions of other questions were mainly driven by the participating clinicians and researchers (Table 2, questions 5 and 7). Other questions received similar prioritization from all participants.

**Table 2:** The pregnancy-associated breast cancer science agenda, as determined by a priority-setting partnership of patients, healthcare professionals and researchers. Questions are ranked in order of prioritization.

No	Question
1	What factors within the physiological environment of pregnancy, including the potential breastfeeding period, enable the growth of both HR positive and HR negative tumors?
2	Which external factors, such as lifestyle, stress, dietary choices, and supplements (e.g., folic acid), influence the development and/or recurrence of pregnancy-associated breast cancer?
3	What are the causes of delayed diagnosis in pregnancy-associated breast cancer?
4	Does pregnancy influence the effectiveness of chemotherapy?
5	How can robust and clinically useful data be obtained regarding the safety of targeted therapies during pregnancy?
6	When is it safe to become pregnant again after pregnancy-associated breast cancer, and does this timing differ across specific tumor subtypes (HR positive/negative, HER2 positive/negative)?
7	Does pregnancy itself provide a protective effect against chemotherapy-induced ovarian damage, and is this effect comparable to that of ovarian suppression used during chemotherapy outside of pregnancy?
8	How do hormonal fluctuations during pregnancy and the subsequent breastfeeding period affect long-term quality of life and the risk of breast cancer recurrence?
9	What are the consequences of pregnancy-associated breast cancer for children born to affected mothers?
10	How does oncological treatment during and after pregnancy influence maternal–infant bonding?

Abbreviations: HR: hormone receptor, HER2: human epidermal growth factor receptor 2.

Next to the research questions, five important signals for improvement of clinical care for PABC patients were identified (Table 3). These function as calls-to-action for both local healthcare policies, but may also aid future guideline recommendations on PABC.

**Table 3:** Signals for improvement of clinical care for PABC patients, as determined by a priority-setting partnership of patients, healthcare professionals and researchers.

Signal 1	There is a need for a continuously updated, easily accessible overview of diagnostic and treatment options and limitations in PABC care, based on the latest evidence (e.g., recommendations from the Advisory Board on Cancer, Infertility and Pregnancy). This overview can be either national or supranational.
Signal 2	A (supra)national multidisciplinary guideline is needed to define the minimum specialist involvement in PABC care, particularly regarding management during pregnancy and future fertility counseling.
Signal 3	It is noted that insufficient PABC awareness among primary care providers (e.g., general- or family medicine practitioners, midwifes et cetera) may contribute to a delayed diagnosis. Therefore, an increase in PABC awareness is recommended.
Signal 4	Specific countries or regions should clearly map psychosocial and practical support services that may be applicable to PABC patients, such as childcare and local patient gatherings. This must be integrated with personalized follow-up care.
Signal 5	Greater attention is needed for the role and support needs of patients' families. Patient participants specifically mentioned the role of their parents, of whom some felt to be marginally involved, as partners of patients usually have a more prominent role in the disease trajectory.

Abbreviations: PABC: pregnancy-associated breast cancer.

## 4 Discussion

This Dutch PSP highlights clinically meaningful gaps in PABC knowledge. Despite growing scientific interest in PABC, many fundamental biological, clinical and psychosocial questions remain unanswered. Principles of existing frameworks, such as the James Lind Alliance Guidebook or the Dialogue Model [25,26], were largely followed in this project, whilst the specific implementation of a Socratic dialogue leader, weighted voting system and inclusion of ‘signals’ besides the research agenda were methodologic additions.

The Socratic dialogue method was chosen because it uses lived experiences of participants as a starting point for collective prioritization, rather than pre-framing topics through expert-driven agendas. The weighted voting system was preferred over equal-weight ranking [25] as it allowed participants to express the relative strength of their priorities, better capturing nuance in a small group setting. We included 'signals' alongside the formal research agenda as some unmet needs are addressable through practice change rather than new research, a distinction meaningful to patients but rarely captured in traditional PSPs. Together, these adaptations aimed to maximize patient involvement while remaining within the broader essence of established PSP frameworks.

In the following sections, we contextualize the identified knowledge gaps by the PSP within the current scientific evidence.

### 4.1 Question 1: physiological drivers of tumor growth during pregnancy and breastfeeding

Pregnancy and lactation are characterized by profound hormonal, immunological, and metabolic changes influencing mammary gland remodeling [[27], [28], [29], [30]], potentially promoting tumor growth across both hormone receptor (HR)-positive and HR-negative subtypes. However, most mechanistic data are derived from animal models or non-PABC populations, and human translational studies remain scarce. Crucially, it is unclear why relatively aggressive phenotypes (i.e., high-grade and HR-negative tumors) are overrepresented in PABC [[8], [9], [10], [11], [12], [13]]. The frequent occurrence of HR-negative tumors therefore suggests additional, non-hormonal or non-classical hormonal drivers that are insufficiently understood.

### 4.2 Question 2: influence of external factors on PABC development and recurrence

Lifestyle factors, stress, dietary patterns, and supplement use (e.g., folic acid and antioxidants) may interact with pregnancy-related biology to influence tumor growth or recurrence risk. While several of these factors have been studied in the general breast cancer population [[31], [32], [33]], evidence specific to PABC is extremely limited. Pregnant patients are often excluded from observational cohorts, and recall bias complicates retrospective analyses. As a result, potential modifiable risk factors in PABC remain largely unknown.

Folic acid supplements, widely used before conception and during pregnancy, especially came up as a discussion point during the PSP sessions, as there is limited research hypothesizing folic acid to be a potential growth factor for neoplastic cells [34,35]. Reassuringly, a meta-analysis shows no increased long-term breast cancer risk in people with high folate or folic acid intake [33]. Nevertheless, the direct influence of relatively high circulating folate levels (for example due to supplement usage) during breast cancer development is not yet known [36].

### 4.3 Question 3: causes of delayed PABC diagnosis

Diagnostic delay is a well-documented contributor to poorer outcomes in (breast) cancer [37,38]. PABC patients may be more prone to diagnostic delay as physiological breast changes during pregnancy and lactation may obscure symptoms, while both patients and clinicians may attribute potential symptoms to ‘normal’ pregnancy-related causes [7]. A Swedish study observed a potentially increased time from symptoms to first healthcare contact for PABC versus matched controls, although this difference was not statistically significant [39]. Despite recognition of this problem, systematic evaluation of the specific causes of delays (e.g., healthcare system factors, healthcare provider-level factors and patient-level factors) is not yet performed, limiting targeted interventions. Yet, lived experiences from multiple participating patients revealed that unawareness did lead to delays in diagnostic referrals.

### 4.4 Question 4: impact of pregnancy on chemotherapy effectiveness

Chemotherapy during the second and third trimesters is considered feasible and relatively safe for the fetus [[40], [41], [42]]. However, whether pregnancy alters pharmacokinetics, pharmacodynamics, or tumor responsiveness, remains unclear. Illustratively, PrBC patients in this PSP indicated that the chemotherapy side effects were milder for cycles during pregnancy in comparison to the postpartum chemotherapeutic cycles. Although cycles accumulate, there is evidence that dose exposure during pregnancy may be lower due to an increased volume of distribution and clearance [43,44], thereby potentially impacting therapy effectiveness. Available studies are small, heterogeneous, and underpowered to detect differences in efficacy by gestational age or tumor subtype. As such, current treatment strategies assume, but have not conclusively demonstrated, equivalent effectiveness.

### 4.5 Question 5: generating robust safety data on targeted therapies

Evidence on the safety of targeted therapies (e.g., anti-HER2 agents, CDK4/6 inhibitors, immunotherapy, PARP inhibitors) during pregnancy is extremely limited and largely based on case reports or inadvertent exposure [[45], [46], [47], [48]]. Obviously, traditional safety trials are not possible in this highly vulnerable patient population. Therefore, collaborative and innovative studies, such as prospective registries, international data pooling, preclinical studies with relevant models, and pharmacovigilance frameworks are urgently needed to support evidence-based counseling for PABC patients. An example in the Netherlands could be the Dutch Melanoma Treatment Registry, a quality register that includes all Dutch melanoma patients who receive targeted- and/or immunotherapy [49], or the extensive quality registers that exist in Sweden [50].

### 4.6 Question 6: timing of subsequent pregnancy after PABC

Studies suggest that pregnancy after breast cancer does not worsen prognosis, even in HR-positive disease [51,52]. Moreover, a recent study in patients with a pathogenic *BRCA* mutation diagnosed with PrBC showed no concerns regarding reproductive outcomes or maternal prognosis [53]. Nevertheless, further PABC-specific data is generally sparse, and subgroup analyses by tumor biology, treatment exposure, and time interval are usually underpowered. For patients, this was an important topic as they experience a strong need for personalized counseling regarding subsequent pregnancies (and fertility) after PABC.

### 4.7 Question 7: Protective effects of pregnancy on ovarian function

In premenopausal women with breast cancer receiving chemotherapy, gonadotropin-releasing hormone agonists (GnRHa) are effective in preserving ovarian function and reducing premature ovarian insufficiency rates [54]. For PrBC patients, it is hypothesized that the pregnancy itself as an ovulation suppressor may theoretically protect against chemotherapy-induced ovarian damage. However, it is not known whether this holds true, and whether this offers comparable protection of ovarian function to GnRHa in non-pregnant premenopausal women. A study on the ovarian reserve of mice treated with various chemotherapeutic agents during pregnancy indeed showed ovarian damage after treatment, although gonadotoxicity differed per agent. Of course, these data are hypothesis-generating and not directly transferable to the human setting [55]. Altogether, data on ovarian reserve and fertility outcomes after treatment for PABC are limited, which precludes informed and evidence-based fertility counseling.

### 4.8 Question 8: Hormonal fluctuations, quality of life, and recurrence risk

The postpartum period, especially after cessation of breast feeding, is characterized by abrupt hormonal changes and involution of the mammary gland. These processes are linked to inflammation and potential tumorigenic alterations in preclinical models [30,56]. Clinically, however, the long-term effects of pregnancy- and lactation-related hormonal changes on quality of life, survivorship, and recurrence risk are poorly characterized. Prospective longitudinal studies integrating endocrine monitoring, cancer outcomes, and validated quality-of-life measures are needed to clarify these associations.

### 4.9 Question 9: Consequences for offspring

Recent research showed that long-term neonatal outcomes following *in utero* chemotherapy exposure (from the second trimester onwards) appear to be similar to non-exposed controls [42], which is especially reassuring for PrBC patients undergoing systemic treatments during pregnancy. In contrast, children of PPBC patients are not systematically included in studies, as these children were not exposed *in utero* to maternal treatments. Therefore, studies evaluating the development of PPBC patients’ offspring, especially for psychosocial outcomes, are warranted to improve PPBC patient counseling.

### 4.10 Question 10: Maternal-infant bonding and psychosocial impact

Cancer diagnosis and treatment during or shortly after pregnancy may influence many aspects of maternal-infant bonding, such as breastfeeding, attachment, and maternal mental health [57]. Despite its importance for both maternal and child wellbeing, this topic remains under-researched, also leading to conflicting advice to patients. In addition, most studies purely focus on medical outcomes, leaving psychosocial aspects underrepresented, thereby limiting the development of supportive interventions.

### 4.11 Overall synthesis

Interestingly, there was a slight disperse between questions prioritized mainly by patients (2, 8, 9 and 10) and by the clinicians/researchers (5 and 7). This effect has been observed before as well in several other fields [58,59], and especially underscores the importance of involving stakeholders from different backgrounds in research prioritization setting. One could argue that is logical for medical oncologists to experience the lack of safety data on targeted treatments during pregnancy as a major challenge in PABC care (question 5), whilst PABC patients themselves may especially have noticed hormonal fluctuations (question 8) whilst being less prone to mention this to their oncology- or obstetric care provider during their anti-cancer treatment.

With this agenda, this PSP hopes to inspire and guide future research of international research groups with specific expertise on one (or more) of the here identified priorities. Given the diverse nature of the research questions, ranging from fundamental biology and clinical observational studies to psychosocial research, we propose establishing a dedicated PABC research community to bring the results of these joint efforts together. The existing infrastructure of the International Network for Cancer, Infertility and Pregnancy (INCIP) registry could, for example, support this initiative, alongside treatment advisory boards such as the Advisory Board on Cancer, Infertility and Pregnancy (ABCIP) and affiliated national bodies, which already bridge clinical and research expertise in this field.

This study represents the first dedicated PABC PSP, aimed at establishing a tailored and relevant science agenda for this patient group. By integrating input of patients, clinicians and researchers, we developed a multidisciplinary agenda as comprehensive as possible. As more patients than clinicians/researchers participated, the agenda naturally skewed towards the topics patients deemed most important.

The main limitation of this project is its limited diversity. First, as this project was held in the Netherlands, this may influence generalizability for PABC patients in other countries or continents. As healthcare in the Netherlands is relatively well-accessible and (almost) fully reimbursed by a mandatory health insurance [60], patients in other countries with differently organized healthcare systems may face other challenges. For example, such a PSP may also prioritize research on access to treatment or financial impact, which was not discussed in our PSP. Although a total of 19 patients were involved in the sounding board and focus group, this relatively limited sample size may have an impact on the robustness of the prioritization, as a larger or more diversely composed group might have weighted certain questions differently. Moreover, our patient population was, despite efforts to reach as many patients as possible through patient associations and through treating clinicians, not highly diverse in socioeconomic status, ethnicity or education level. Patients from other socioeconomic backgrounds or minority ethnic groups may face distinct barriers and concerns that are underrepresented in the current agenda. This is of course a major limitation in many patient participation projects [61], and we therefore recommend further validation of this agenda in other countries and with a more diverse patient representation.

### 4.12 Conclusions

This PSP identified ten key research questions that address critical biological, clinical, and psychosocial uncertainties in PABC, in addition to five signals to improve clinical care. Despite increasing clinical experience and improving maternal and fetal outcomes, current evidence remains fragmented, retrospective, and insufficiently tailored to the unique physiological context of pregnancy and the postpartum period. The identified priorities underscore the need for integrative research approaches that combine mechanistic studies, innovative observational designs, and long-term follow-up of both mothers and children. Addressing these gaps requires international collaboration, harmonized data collection, and the inclusion of diverse patient perspectives throughout the research process.

## CRediT authorship contribution statement

**Carsten F.J. Bakhuis:** Writing – original draft, Project administration, Methodology, Investigation, Funding acquisition, Formal analysis, Data curation, Conceptualization. **Anna S. Koning:** Writing – review & editing, Investigation. **Britt B.M. Suelmann:** Writing – review & editing, Investigation. **Inge O. Baas:** Writing – review & editing, Investigation. **Janna W. Nijkamp:** Writing – review & editing, Investigation. **Kirsten Coutinho-Steentjes:** Writing – review & editing, Investigation. **Pleun A. Gilsing-Doreleijers:** Writing – review & editing, Investigation. **Manuela Guerra:** Writing – review & editing, Investigation. **Aaltje G.H. Hoppentocht:** Writing – review & editing, Investigation. **Elsemieke Kaashoek-de Jong:** Writing – review & editing, Investigation. **Nicol Nijholt:** Writing – review & editing, Investigation. **Roselien van der Wielen:** Writing – review & editing, Investigation. **Jan Ewout Ruiter:** Writing – review & editing, Investigation. **Sabine C. Linn:** Writing – review & editing, Investigation. **Stefan Preković:** Writing – original draft, Investigation. **Paul J. Van Diest:** Writing – original draft, Investigation. **Elsken van der Wall:** Writing – original draft, Investigation. **Carmen van Dooijeweert:** Writing – review & editing, Writing – original draft, Project administration, Methodology, Investigation, Funding acquisition, Formal analysis, Data curation, Conceptualization.

## Ethical approval

This study was approved by the institutional research quality coordinator, ensuring our compliance with legislation and regulations (No. 24U-1805). This study did not fall under the scope of the Dutch Medical Research Involving Human Subjects Act (WMO) and therefore did not require further approval from an accredited MREC in the Netherlands. All participating patients in the focus group provided written informed consent.

## Data statement

All research data of this project has been included in the manuscript and associated tables and supplemental tables. Transcripts and notes of the focus group meetings are securely stored and not available for retrieval due to privacy constraints. However, the here described results are a complete reflection of the content and results of the several sessions.

## Declaration of competing interests statement

CFJB has received institutional research funding from Gilead Sciences (this project) and (speaker) honoraria from Congress Care and Congress & Meeting Services Holland.

BBMS has an advisory role for AstraZeneca, Bristol Myers Squibb, MDS, IPSEN, Merck, Astellas and Pfizer (institutional) and has received research support from AstraZeneca, Bristol Myers Squibb, MDS, IPSEN, Merck, Astellas and Pfizer (institutional).

SCL received institutional research support from Agendia, Amgen, AstraZeneca, Eurocept, Genentech, Immunomedics (now Gilead Sciences), Roche, Sanofi, and TESARO (now GSK). SCL serves on the advisory board for AstraZeneca, with compensation paid to the institute, and for Cergentis on a pro bono basis. SCL also receives funding for educational activities from Daiichi Sankyo, paid to the institute.

PvD is a member of the advisory boards of Visiopharm, Paige and Sectra.

CvD received institutional research funding from Gilead Sciences (this project), Visiopharm, Paige and Ellogon. AI, and (speaker) honoraria from Benecke, Congress Care and IQVIA.

All other authors report no conflicts of interest.
