# Supplementary material for: Towards patient-centered research in pregnancy-associated breast cancer: creating a science agenda through a priority-setting-partnership
Source: Breast. 2026 May 15;88:104808. doi: 10.1016/j.breast.2026.104808 (PMC13213276; doi:10.1016/j.breast.2026.104808)
Supplement: Multimedia component 1 [file mmc1.docx]

**SUPPLEMENTAL TABLES**

| **Number** | **Question** |
| --- | --- |
| 1 | What factors within the physiological environment of pregnancy, including the potential breastfeeding period, enable the growth of both HR positive and HR negative tumors? |
| 2 | Which external factors, such as lifestyle, stress, dietary choices, and supplements (e.g., folic acid), influence the development and/or recurrence of pregnancy-associated breast cancer? |
| 3 | Does breastfeeding have an impact on the risk of recurrence of pregnancy-associated breast cancer? |
| 4 | What are the causes of delayed diagnosis in pregnancy-associated breast cancer? |
| 5 | Does pregnancy influence the effectiveness of chemotherapy? |
| 6 | How can robust and clinically useful data be obtained regarding the safety of targeted therapies during pregnancy? |
| 7 | Is there a difference in side effects and impact on quality of life when using antihormonal therapy after pregnancy-associated breast cancer, compared to non-pregnancy-associated breast cancer? |
| 8 | When is it safe to become pregnant again after pregnancy-associated breast cancer, and does this timing differ across specific tumor subtypes (HR positive/negative, HER2 positive/negative)? |
| 9 | Does pregnancy itself provide a protective effect against chemotherapy-induced ovarian damage, and is this effect comparable to that of ovarian suppression used during chemotherapy outside of pregnancy? |
| 10 | How do hormonal fluctuations during pregnancy and the subsequent breastfeeding period affect long-term quality of life and the risk of breast cancer recurrence? |
| 11 | What causes weight gain after treatment for pregnancy-associated breast cancer? |
| 12 | What are the consequences of pregnancy-associated breast cancer for children born to affected mothers? |
| 13 | How does oncological treatment during and after pregnancy influence maternal-infant bonding? |
| 14 | How does treatment during and after pregnancy affect the relationship with your partner/loved ones? |

**Table S1**. Overview of all curated research questions, in random order, that were prioritized by the participants to form the definitive top-10 science agenda.
*Abbreviations: HR: hormone receptor, HER2: human epidermal growth factor receptor 2.*

| **Question** | | **Total tokens** | **Patients** | **HCPs** | **Researchers** |
| --- | --- | --- | --- | --- | --- |
| **1** | Internal factors | 48 | 29 | 10 | 9 |
| **2** | External factors | 30 | 28 | 0 | 2 |
| **3** | Breast feeding | 14 | 8 | 2 | 4 |
| **4** | Delayed diagnosis | 17 | 9 | 7 | 1 |
| **5** | Therapy effectiveness | 31 | 14 | 10 | 7 |
| **6** | Targeted therapies | 29 | 7 | 15 | 7 |
| **7** | Antihormonal therapy | 11 | 10 | 0 | 1 |
| **8** | Subsequent pregnancy | 24 | 12 | 5 | 7 |
| **9** | Fertility preservation | 14 | 4 | 5 | 5 |
| **10** | Hormonal fluctuations | 19 | 19 | 0 | 0 |
| **11** | Weight gain | 5 | 5 | 0 | 0 |
| **12** | Children | 17 | 14 | 1 | 2 |
| **13** | Infant bonding | 16 | 12 | 4 | 0 |
| **14** | Partner/loved ones | 10 | 9 | 1 | 0 |

**Table S2**. Overview of token distribution per research question (similar order as in Supplemental Table S1), subdivided by patient tokens, healthcare providers and researchers.
*Abbreviation: HCPs: healthcare providers.*
